# Use of hidden Markov capture–recapture models to estimate abundance in the presence of uncertainty: Application to the estimation of prevalence of hybrids in animal populations

**DOI:** 10.1002/ece3.4819

**Published:** 2019-02-05

**Authors:** Nina Luisa Santostasi, Paolo Ciucci, Romolo Caniglia, Elena Fabbri, Luigi Molinari, Willy Reggioni, Olivier Gimenez

**Affiliations:** ^1^ Department of Biology and Biotechnologies “Charles Darwin” University of Rome La Sapienza Rome Italy; ^2^ CEFE, CNRS University of Montpellier, University Paul Valéry Montpellier 3, EPHE, IRD Montpellier France; ^3^ Italian Institute for Environmental Protection and Research (ISPRA) Unit for Conservation Genetics (BIO‐CGE) Ozzano dell'Emilia Bologna Italy; ^4^ Wolf Apennine Center Appennino Tosco‐Emiliano National Park Ligonchio Italy

**Keywords:** anthropogenic introgression, capture–recapture, hidden Markov models, hybridization, multievent models, prevalence, Viterbi algorithm

## Abstract

Estimating the relative abundance (prevalence) of different population segments is a key step in addressing fundamental research questions in ecology, evolution, and conservation. The raw percentage of individuals in the sample (naive prevalence) is generally used for this purpose, but it is likely to be subject to two main sources of bias. First, the detectability of individuals is ignored; second, classification errors may occur due to some inherent limits of the diagnostic methods. We developed a hidden Markov (also known as multievent) capture–recapture model to estimate prevalence in free‐ranging populations accounting for imperfect detectability and uncertainty in individual's classification. We carried out a simulation study to compare naive and model‐based estimates of prevalence and assess the performance of our model under different sampling scenarios. We then illustrate our method with a real‐world case study of estimating the prevalence of wolf (*Canis lupus*) and dog (*Canis lupus familiaris*) hybrids in a wolf population in northern Italy. We showed that the prevalence of hybrids could be estimated while accounting for both detectability and classification uncertainty. Model‐based prevalence consistently had better performance than naive prevalence in the presence of differential detectability and assignment probability and was unbiased for sampling scenarios with high detectability. We also showed that ignoring detectability and uncertainty in the wolf case study would lead to underestimating the prevalence of hybrids. Our results underline the importance of a model‐based approach to obtain unbiased estimates of prevalence of different population segments. Our model can be adapted to any taxa, and it can be used to estimate absolute abundance and prevalence in a variety of cases involving imperfect detection and uncertainty in classification of individuals (e.g., sex ratio, proportion of breeders, and prevalence of infected individuals).

## 1 INTRODUCTION

The relative abundance (prevalence) of different population segments is a fundamental piece of information to understand processes in ecology, evolution, and conservation. For example, the prevalence of infected individuals is critical to understand the mechanisms driving disease dynamics (Jennelle, Cooch, Conroy, & Senar, 2007; Moreno‐Torres, Wolfe, Saville, & Garabed, 2016); the prevalence of key demographic categories (e.g., mature females) is needed to assess the viability of endangered populations (Caswell, 2000) and when hybridization represents a threat, the prevalence of admixed individuals is needed to evaluate the appropriate management option (Allendorf, Leary, Spruell, & Wenburg, 2001).

However, estimating prevalence for wildlife populations is challenging and the raw percentage of individuals of a class in the sample (naive prevalence) is often used as a proxy (Jennelle et al., 2007). However, this approach overlooks two main sources of bias. First, imperfect and/or heterogeneous detection leads to biased abundance estimates when it is ignored (Cubaynes et al., 2010; Jennelle et al., 2007). Second, uncertainty in the classification of individuals (e.g., diseased/healthy, breeder/nonbreeder, male/female) is common in wildlife population studies where individuals are assigned to a specific status based on imperfect clues. Examples include determining sex or breeding status based on the behavior of individuals (Genovart, Pradel, & Oro, 2012) or establishing health status from the observation of outer symptoms only (Conn & Cooch, 2009). Another less explored but intriguing situation is assigning individuals to genetic classes (subpopulations) based on a limited number of genetic markers (Vähä & Primmer, 2006).

Reliable estimates of wildlife abundance can be obtained by correcting field counts by the proportion of undetected individuals (i.e., the ratio between the number of observed individuals and the probability of detection; Nichols, 1992). The probability of detection is usually estimated by using capture–recapture models (CR) from a sample of individual encounter histories (Otis, Burnham, White, & Anderson, 1978). In particular, multistate CR models estimate the probability of detection for different classes of individuals by assigning individuals to static or dynamic states. However, multistate CR models assume the correct assignment of all individuals to their state (Lebreton, Burnham, Clobert, & Anderson, 1992). Multievent models relax this assumption by acknowledging the uncertainty of the observation process in the model structure (Pradel, 2005). In these models, a hidden biological process (e.g., survival or dispersal) is modeled as a Markov chain of states (Pradel, 2005). The observation process (the data) arises from the underlying states through the probability of detection. To include uncertainty in state assignment, the observation process is further split into two steps: detection and state assignment conditional on detection (Gimenez, Lebreton, Gaillard, Choquet, & Pradel, 2012; Pradel, 2005). This formulation includes a probability of assignment (besides the probability of detection and the probabilities associated with the biological process), allowing for the inclusion of individuals classified with uncertainty (Pradel, 2005).

Multievent models have been used to estimate a variety of population parameters in the presence of uncertainty in state assignment. Examples of that include the rates of entry and exit from disease states (Conn & Cooch, 2009), the probability of skipping reproduction (Sanz‐Aguilar et al., 2011), and the probability of survival of different age classes (Gervasi et al., 2017). However, multievent models have never been used to estimate the abundance of individuals in different states because the numerator of the abundance estimator (the number of observed individuals) is contaminated by uncertain observations.

Here, we develop a capture–recapture approach to estimate the prevalence of admixed individuals (hereafter “hybrids”) in a population while simultaneously accounting for both imperfect detection and classification uncertainty. Specifically, we show how to use the multievent CR framework to estimate abundance of individuals in different states (i.e., “Parental,” “Hybrid,” “Dead”) in the presence of uncertainty in state assignment. We first use multievent models to estimate survival and detection parameters; second, we use the Viterbi algorithm to assign the uncertain observed individuals to the most likely state (Rouan, Gaillard, Guédon, & Pradel, 2009; Zucchini, MacDonald, & Langrock, 2009), and lastly, we estimate prevalence via a Horvitz–Thompson‐like estimator combined with a bootstrapping procedure to produce standard error and confidence intervals (Davison & Hinkley, 1997).

We assess the importance of incorporating detectability and uncertainty in state assignment by comparing the performance of model‐based and naive prevalence under different scenarios. The accuracy of CR parameters’ estimators depends on the recapture rate and on the number of capture occasions (Otis et al., 1978). Increasing the detectability and/or the number of occasions requires different sampling strategies and generates different costs in terms of financial and human resources (Lieury et al., 2017). Therefore, we also explore how different sampling strategies may affect the model performance.

Despite the increasing attention that researchers are devoting to hybridization cases (Schwenk, Brede, & Streit, 2008; Todesco et al., 2016), there have been only few attempts to estimate prevalence of hybrids in wild populations (Vaz Pinto, Beja, Ferrand, & Godinho, 2015). We illustrate our method with a case study by estimating the prevalence of wolf (*Canis lupus*) × dog (*Canis lupus familiaris*) hybrids in a wolf population in northern Italy. This is a case of anthropogenic hybridization (i.e., the interbreeding of individuals from genetically distinct populations due to human action; Allendorf et al., 2001) and is considered a major threat to wolf genomic integrity (Boitani, 2000). Therefore, accurately estimating the prevalence of hybrids in wolf populations is a priority to elaborate conservation strategies (Hindrickson et al., 2017). We show that in this case using naive prevalence as a proxy underestimates the prevalence of hybrids.

## 2 METHODS

### 2.1 Hidden Markov model

We assumed that animals are individually recognized at discrete encounter occasions, therefore obtaining an encounter history for each individual. Individuals can be in one of three possible nonobservable states: alive and parental (P), alive and hybrid (H), or dead (D). We underline that hereafter the term “hybrid” refers to all categories of admixture and not only to first‐generation hybrids. Upon its first encounter, an individual has a probability π_*p*_ to be a parental and the complementary probability π_*h*_ = 1 − π_*p*_ to be a hybrid. The initial state probabilities describe the probability that an individual is in one or another state when first encountered. Then, the states change over time according to a first‐order Markov process, with the state process being governed by apparent survival probabilities *φ*_*p*_ and *φ*_*h*_. More specifically, the state process, which summarizes the underlying biological process, is represented by a transition probability matrix with states at time *t* in rows (“Parental,” “Hybrid,” “Dead”) and *t *+* *1 in columns (“Parental,” “Hybrid,” “Dead”):

$$\begin{array}{c} \begin{array}{c} \begin{array}{cccc} & P & H & D \end{array}\\ \begin{array}{c} p\\ H\\ D \end{array}\left(\begin{array}{ccc} \varphi_{p} & 0 & 1-\varphi_{p}\\ 0 & \varphi_{h} & 1-\varphi_{h}\\ 0 & 0 & 1 \end{array}\right) \end{array} \end{array}$$

where parameter *φ*_*p*_ (resp. *φ*_*h*_) is the probability that an individual alive and in state “Parental” (resp. “Hybrid”) at time *t* is still alive in the study area and in state “Parental” (resp. “Hybrid”) at time *t *+* *1 and corresponds to the apparent survival probability of parental (resp. “Hybrid”) individuals.

The second time series (or event process) is generated from the states at each occasion and describes the observation process. Individuals are detected at time *t* with probability of detection *p*_*p*_ for parental and *p*_*h*_ for hybrid individuals. Upon detection, an attempt is made of assigning the individuals to a hybridization state based on genetic and/or morphologic diagnostic features and there is a probability *δ* that an individual is assigned to the state “Parental” or “Hybrid”. If the diagnostic clues are not sufficient to ascertain the hybridization state, the individual is classified as uncertain with the complementary probability 1−*δ*. The observation process is summarized by a matrix with states in rows (“Parental,” “Hybrid,” “Dead”) and events in columns (0 = “Not detected,” 1 = “Detected as parental,” 2 = “Detected as hybrid,” 3 = “Detected as uncertain”):

$$\begin{array}{c} \begin{array}{c} \begin{array}{ccccc} & 0\; & 1\; & 2\; & 3 \end{array}\\ \begin{array}{c} p\\ H\\ D \end{array}\left(\begin{array}{cccc} 1-p_{p} & p_{p}\delta_{p} & 0 & p_{p}\left(1-\delta_{p}\right)\\ 1-p_{h} & 0 & p_{h}\delta_{h} & p_{h}\left(1-\delta_{h}\right)\\ 1 & 0 & 0 & 0 \end{array}\right). \end{array} \end{array}$$

In the first row, the (1 − *p*_*p*_) term is the probability that an individual in state “Parental” is not detected, and *p*_*h*_ *δ*_*h*_ is the probability that an individual in state “Parental” is detected and assigned to the category “Parental” while *p*_*p*_(1 − *δ*_*p*_) is the probability that an individual in state “Parental” is detected (*p*_*p*_) and not assigned to any category (1 − *δ*_*p*_). Note that an individual in state “Parental” cannot be detected as a hybrid; hence, the corresponding probability is 0. The second row is similar to the first one, except that it refers to the hybrid individuals. In the third row, all individuals are nondetected because they are dead. Equivalently, the event process can be decomposed as the product of a detection matrix by an assignment matrix which expresses the probability that an individual is assigned to a state given that it has been detected:

$$\begin{array}{c} \begin{array}{ccc} & \begin{array}{cccc} & \;0 & \;\;1 & 2\; \end{array} & \begin{array}{ccccc} & \;\;0\; & 1\; & 2\; & 3\;\;\; \end{array}\\ & \begin{array}{c} p\\ H\\ D \end{array}\left(\begin{array}{ccc} 1-p_{p} & p_{p} & 0\\ 1-p_{h} & 0 & p_{h}\\ 1 & 0 & 0 \end{array}\right) & \begin{array}{c} 0\\ 1\\ 2 \end{array}\left(\begin{array}{cccc} 1 & 0 & 0 & 0\\ 0 & \delta_{p} & 0 & 1-\delta_{p}\\ 0 & 0 & \delta_{h} & 1-\delta_{h} \end{array}\right) \end{array} \end{array}$$

when an animal is first encountered, the capture process is not modeled because an animal must be encountered at least once to enter the dataset, but the state ascertainment remains valid.

To illustrate the calculation of an encounter history, let us consider the case of a 3‐year CR experiment. For instance, the encounter history “303” denotes an individual encountered at the first and third occasions but not at the second occasion. The state of this individual is never assigned. Assuming parameters are constant, we have

$$\begin{array}{cc} \mathrm{Pr}\left(303\right) & =\pi_{h}\left(1-\delta_{h}\right)\varphi_{h}\left(1-p_{h}\right)\varphi_{h}p_{h}\left(1-\delta_{h}\right)\\ & \;+\pi_{p}\left(1-\delta_{p}\right)\varphi_{p}\left(1-p_{p}\right)\varphi_{p}p_{p}\left(1-\delta_{p}\right) \end{array}$$

On the right side of the equation, the first element of the sum is the probability of the observed history if the underlying state is “Hybrid,” while the second element is the probability of the observed history given that the underlying state is “Parental.” The likelihood of the entire dataset is obtained as the product of the probabilities of all individual encounter histories assuming independence. In this paper, estimates of initial states (π_*p*_ and π_*h*_), apparent survival (*φ*_*p*_ and *φ*_*h*_), detection (*p*_*p*_ and *p*_*h*_), and assignment probabilities (*δ*_*p*_ and *δ*_*h*_) are obtained by maximizing the likelihood function. Different models can be built by allowing the parameters to vary according to, for example, state, time, or group of individuals classified based on discrete variables (e.g., age class or sex).

### 2.2 Abundance and prevalence estimation

Naive prevalence (*P*_naive_) at a given occasion is calculated as the proportion of observed hybrids in the sample:

$$P_{\mathrm{naive}}=\frac{n_{h}}{n_{p}+n_{h}}$$

where *n*_*h*_ is the number of observed hybrids and *n*_*p*_ is the number of observed parentals.

Here, we propose a model‐based prevalence estimate (*P*_model_) as the ratio between hybrids’ abundance in the population and total population abundance. Assuming that marked and unmarked individuals have the same probability of detection, total abundance at a given occasion (${\hat{N}}_{\mathrm{tot}}$) is then estimated with the Horvitz–Thompson estimator (McDonald & Amstrup, 2001) as:

$${\hat{N}}_{\mathrm{tot}}=\frac{n_{p}}{{\hat{p}}_{p}}+\frac{n_{h}}{{\hat{p}}_{h}}={\hat{N}}_{p}+{\hat{N}}_{h}$$

where *n*_*h*_ is the number of observed hybrids, and *n*_*p*_ is the number of observed parental individuals, ${\hat{p}}_{h}$ is the recapture probability of hybrids, and ${\hat{p}}_{p}$ is the recapture probability of parental individuals. Model‐based prevalence is then estimated as:

$$P_{\mathrm{model}}=\frac{{\hat{N}}_{h}}{{\hat{N}}_{p}+{\hat{N}}_{h}}.$$

The main difficulty is therefore in determining the number of observed parental individuals and hybrids, because the uncertain individuals have to be assigned to one of the two states (“Hybrid” or “Parental”). To do so, we use the Viterbi algorithm (Zucchini et al., 2009) which, given any observation sequence (here, the encounter histories) and the parameters estimated by the hidden Markov model, finds the most probable underlying sequence of states that has generated the observed data (Rouan et al., 2009). Once the uncertain observations are assigned to the most likely state, the number of observed hybrid and parental individuals can be reconstructed and the formula can be used to estimate prevalence. We obtain confidence intervals for the hybrids’ prevalence by using a nonparametric bootstrap procedure (Davison & Hinkley, 1997).

### 2.3 Evaluation of model performance and sampling strategy

To test the model performance, we generated encounter histories with known prevalence mimicking our case study on wolves and dogs in the Northern Apennines (see next section) and compared model‐based prevalence and naive prevalence. Using the R (R Core Team, 2017) package HMM (Himmelmann, 2010), we simulated a cohort of 100 individuals that we split into 2 states “Hybrid” and “Parental”. For all the scenarios, we set the initial proportion of wolves π_*p*_ = 0.7 and initial proportion of hybrids π_*h*_ = 1 − π_*p*_ = 0.3 as in our case study sample (see next section). Using values estimated by Caniglia et al. (2012) for the same wolf population, we considered state‐dependent survival (constant over time) with parental survival (φ_*p*_ = 0.8) higher than hybrid survival (φ_*h*_ = 0.7). We then considered three hypothetical scenarios for detectability and assignment probability: (see Supporting information Table S1 for a complete list of parameters for the three scenarios) (1) state‐dependent detectability (*p*_*p*_ > *p*_*h*_) and homogeneous assignment probability, (2) homogeneous detectability and state‐dependent assignment probability (*δ*_*p*_ > *δ*_*h*_) and (3) homogeneous detectability and assignment probability. Within these three main scenarios, we evaluated the effect of lower and higher sampling intensities by comparing sampling strategies with low and high detectability and with 5 and 10 capture occasions. We simulated 100 datasets for each combination of parameters within the three main scenarios. We fitted constant and state‐dependent models to the simulated data, and we calculated the relative bias and the root mean squared error (RMSE) for the model‐based and the naive prevalence estimators. We also calculated the confidence interval coverage for the model‐based estimator of prevalence.

### 2.4 Case study

We collected fresh wolf scats in the Appennino Tosco‐emiliano National Park from August 2016 to May 2017. We extracted, amplified and sequenced DNA from the scats following the procedures described in Caniglia, Fabbri, Galaverni, Milanesi, and Randi (2014). We identified wolves, dogs and putative hybrids based on the analysis of molecular markers listed in Randi et al. (2014) and using Bayesian genetic clustering procedures implemented in STRUCTURE 2.3.4 (Falush, Stephens, & Pritchard, 2003; Pritchard, Stephens, & Donnelly, 2000). We distinguished wolves, hybrids, and uncertain individuals based on their membership proportions to the wolf cluster (*q*_wolf_) and 90% Bayesian credible intervals (BCI). We set the thresholds for the three categories based on the genetic clustering analyses performed on simulated genotypes by Pacheco et al. (2017), see Fig. S1 in the Supplementary Materials of Pacheco et al. (2017). We considered as pure wolves those individuals whose *q*_*wolf*_ was included in the range of *q*_wolf_ values of simulated pure wolves genotypes and did not overlap with that of simulated backcrosses (hybrid × parental). We classified as uncertain those individuals whose *q*_wolf_ was included in the range in which the *q*_wolf_ values of simulated pure wolves and backcrosses to wolves (first and second generation) overlapped. Finally, we considered as hybrids those individuals whose *q*_wolf_ overlapped with the range of *q*_wolf_ values of simulated backcrosses (first and second generation) and/or hybrids (first and second generation). We additionally considered as hybrids those individuals which presented a Y haplotype of canine origin or the deletion at the K‐locus (Caniglia et al., 2013; Randi et al., 2014) regardless of their *q*_wolf_ values.

The CR data were pooled in 2‐month capture occasions, with a total of five capture occasions. Thirty‐nine individuals were sampled (19 wolves, 12 hybrids, 8 uncertains based on their *q*_wolf_ value). We applied the multievent CR model described above to test for differences in detectability and assignment probability between hybrid and parental individuals and to estimate prevalence of wolf × dog hybrids in the population. Since the hybridization assessment is performed only once for each genotype, we constrained the assignment probability to be estimated only upon first capture (see the models details in the Supporting information). We fitted models with state‐dependent and constant parameters and a combination of the two. The models were fitted in the E‐SURGE software (Choquet, Rouan, & Pradel, 2009) and the Viterbi algorithm was implemented using the R (R Core Team, 2017) package HMM (Himmelmann, 2010). We used the Akaike Information Criterion (AIC) for model selection, considering models within *Δ*AICc ≤ 2 as the most supported and used model averaging to account for uncertainty in model selection (Burnham & Anderson, 2002).

## 3 RESULTS

### 3.1 Evaluation of model performance

**Figure 1:**
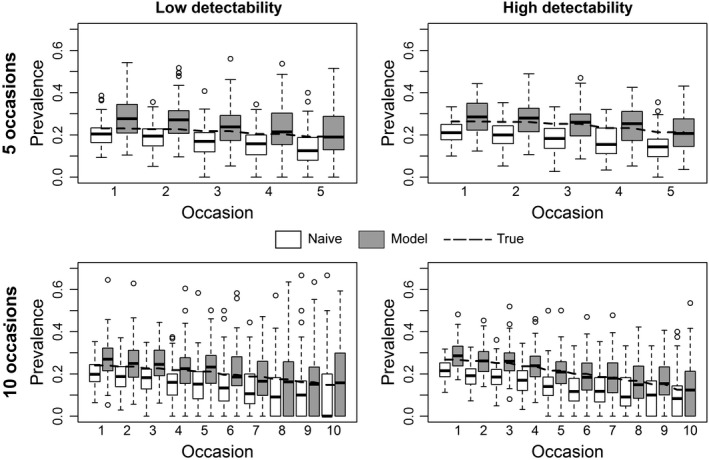
Scenario 1 of simulation (state‐dependent detectability and homogeneous assignment probability). Sampling strategies with 5 (upper panels) versus 10 (lower panels) capture occasions and low (left‐column panels) versus high (right‐column panel) detectability. True prevalence is represented as a dashed line while the 100 values of naive and model‐based prevalence are displayed in the white and gray boxplots, respectively

Naive prevalence had higher RMSE and percent relative bias than model‐based prevalence in scenario 1 (state‐dependent detectability and homogeneous assignment probability; Figure 1, Tables 1, 2). The same occurred in scenario 2 (homogeneous detectability and state‐dependent assignment probability; Supporting information Figure S1 and Tables S2, S3). Naive and model‐based prevalence had similar RMSE and relative bias only in scenario 3 (homogeneous detectability and assignment probability; Supporting information Figure S1 and Tables S4, S5).

**Table 1:** Scenario 1 of simulation (state‐dependent detectability and homogeneous assignment probability)

	Occ.1	Occ.2	Occ.3	Occ.4	Occ.5
**Root mean squared error**					
Low detectability					
Naive	0.07	0.14	0.23	0.23	0.30
Model‐based	0.30	0.15	0.06	0.07	0.02
High detectability					
Naive	0.26	0.36	0.48	0.41	0.48
Model‐based	0.04	0.02	0.00	0.01	0.00
**Percent relative bias**					
Low detectability					
Naive	−0.03	−0.04	−0.05	−0.05	−0.05
Model‐based	0.05	0.04	0.03	0.03	0.01
High detectability					
Naive	−0.05	−0.06	−0.07	−0.06	−0.07
Model‐based	0.02	0.01	0.00	0.01	0.00

Note

Root mean squared error and relative bias of naive and model‐based prevalence for sampling strategies with 5 capture occasions (Occ).

**Table 2:** Scenario 1 of simulation (state‐dependent detectability and homogeneous assignment probability)

	Occ.1	Occ.2	Occ.3	Occ.4	Occ.5	Occ.6	Occ.7	Occ.8	Occ.9	Occ.10
**Root mean squared error**										
Low detectability										
Naive	0.14	0.22	0.21	0.33	0.33	0.30	0.40	0.36	0.26	0.78
Model‐based	0.1	0.04	0.05	0.01	0.01	0.00	0.00	0.00	0.02	0.02
High detectability										
Naive	0.23	0.51	0.37	0.45	0.49	0.50	0.39	0.36	0.16	0.16
Model‐based	0.00	0.00	0.00	0.00	0.00	0.00	0.00	0.00	0.00	0.00
**Percent relative bias**										
Low detectability										
Naive	−0.04	−0.05	−0.05	−0.06	−0.06	−0.05	−0.06	−0.06	−0.05	−0.03
Model‐based	−0.03	−0.02	−0.02	−0.01	−0.01	−0.00	−0.00	−0.01	−0.01	−0.01
High detectability										
Naive	−0.05	−0.07	−0.06	−0.07	−0.07	−0.07	−0.06	−0.06	−0.04	−0.04
Model‐based	0.01	0.00	0.01	0.00	0.01	0.01	0.00	0.01	0.01	0.00

Note

Root mean squared error and relative bias of naive and model‐based prevalence for sampling strategies with 10 capture occasions (Occ).

**Figure 2:**
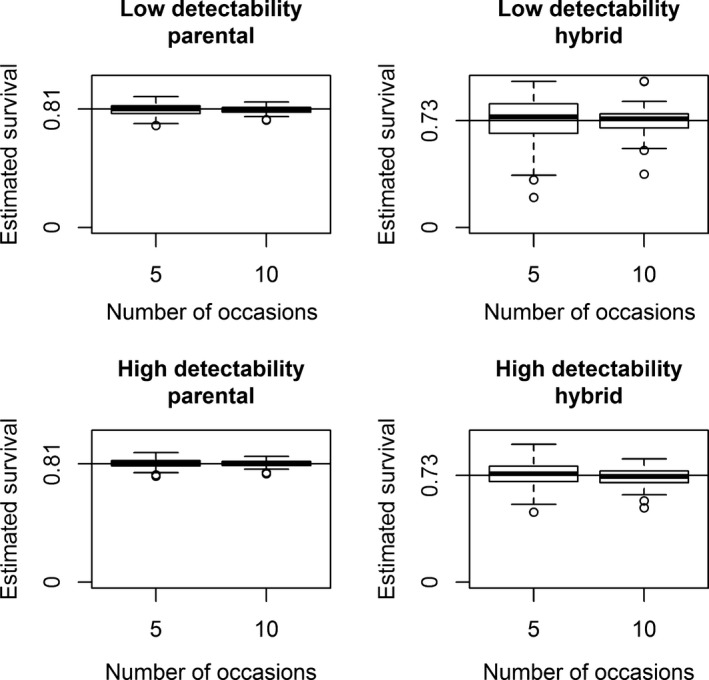
Scenario 1 of simulation (state‐dependent detectability and homogeneous assignment probability). Boxplots of 100 simulated survival estimates for parentals (left two panels) and hybrids (right two panels) for each sampling strategy. Sampling strategies with low detectability are in the top row, and sampling strategies with high detectability are in the bottom row. Estimates obtained with sampling strategies with 5 and 10 capture occasions are compared in each panel

**Figure 3:**
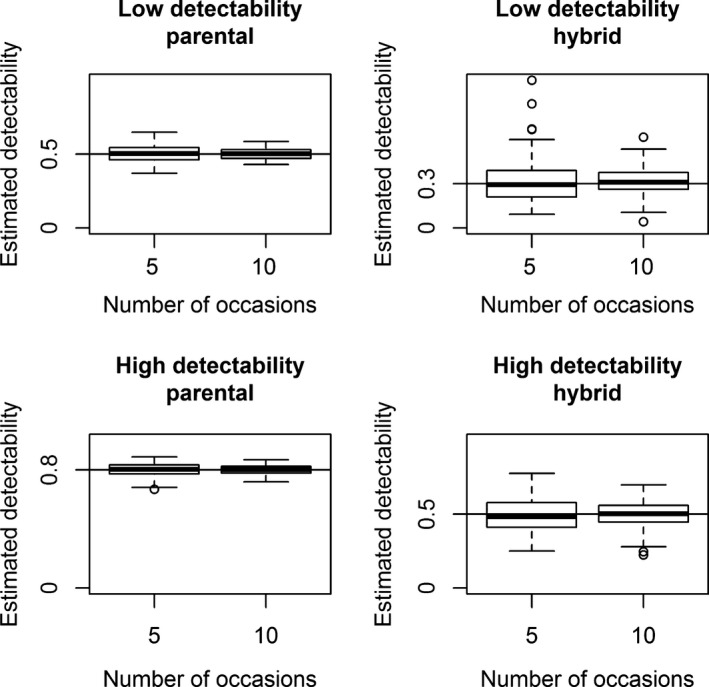
Scenario 1 of simulation (state‐dependent detectability and homogeneous assignment probability). Boxplots of 100 simulated detectability estimates for parentals (left two panels) and hybrids (right two panels) for each sampling strategy. Sampling strategies with low detectability are in the top row, and sampling strategies with high detectability are in the bottom row. Estimates obtained with sampling strategies with 5 and 10 capture occasions are compared in each panel

**Figure 4:**
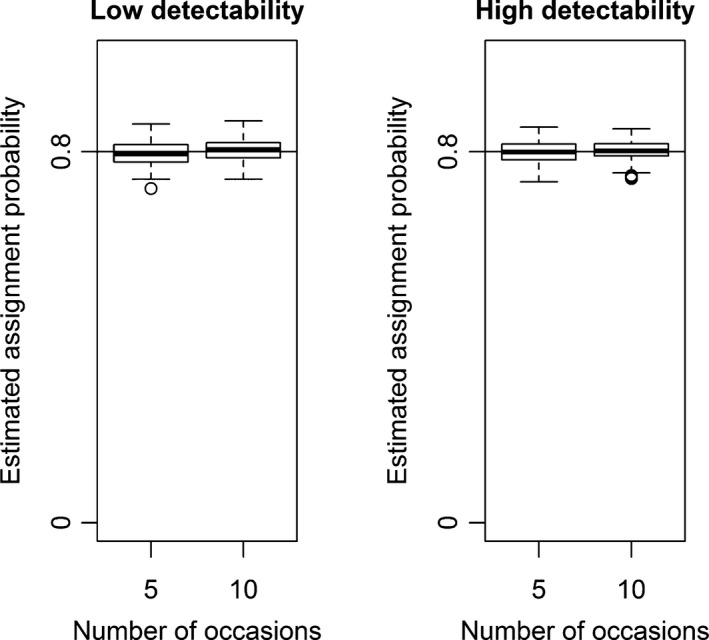
Scenario 1 of simulation (state‐dependent detectability and homogeneous assignment probability). Boxplots of 100 simulated assignment probability estimates. Sampling strategies with low detectability are on the left panel, and sampling strategies with high detectability are on the right panel. Estimates obtained with sampling strategies with 5 and 10 capture occasions are compared in each panel

**Figure 5:**
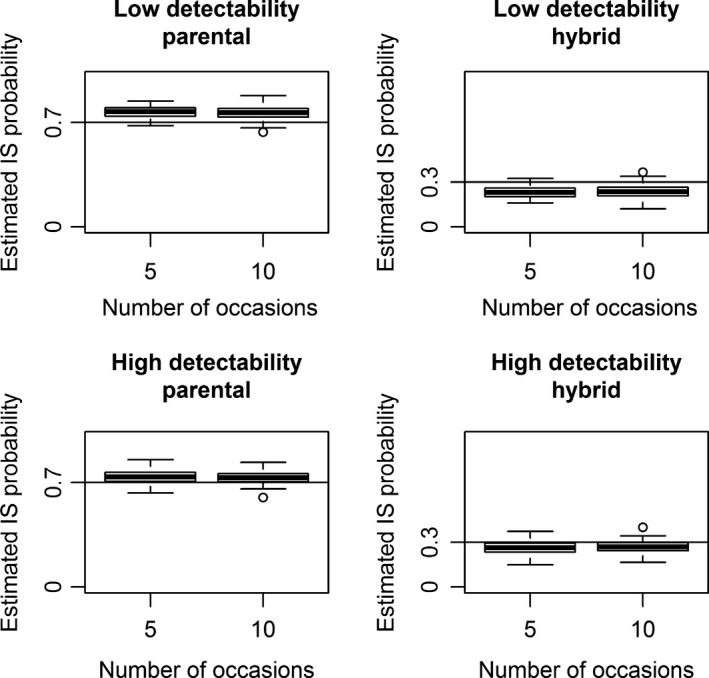
Scenario 1 of simulation (state‐dependent detectability and homogeneous assignment probability). Boxplots of 100 simulated initial state probabilities estimates for parentals (left two panels) and hybrids (right two panels) for each sampling strategy. Sampling strategies with low detectability are in the top row, and sampling strategies with high detectability are in the bottom row. Estimates obtained with sampling strategies with 5 and 10 capture occasions are compared in each panel

The bias associated with model‐based prevalence tended to 0 in scenarios with high detectability (Tables 1, 2; Supporting information Tables S2, S5). Interestingly, the bias associated with naive prevalence had the opposite behavior, as it increased at higher detectability (Tables 1, 2; Supporting information Tables S2–S5). The negative bias in naive prevalence is due to the simulation settings for the true detection and assignment probabilities, and in particular to the fact that *p*_*p*_ > *p*_*h*_ for scenario 1 and *δ*_*p*_ > *δ*_*h*_ for scenario 2. Switching the true values for detectability in scenario 1 and for assignment probability in scenario 2 would cause naive prevalence to be positively‐biased. The bias associated to the estimates of apparent survival, detectability, and probability of assignment was negligible for all scenarios (Figures 2, 3, 4; Supporting information Figures S3–S9), while the estimates of the initial state probability were slightly biased for scenario 1 (Figure 5). Estimates of parameters for the category with fewer individuals (in this case, the hybrids) were less precise, as showed by the boxplot larger ranges (Figures 2, 3, 4; Supporting information Figures S2–S10).

**Table 3:** Scenario 1 of simulation (state‐dependent detectability and homogeneous assignment probability)

Confidence interval coverage 5 occasions						
	Occ. 1	Occ. 2	Occ. 3	Occ. 4	Occ. 5	Average
Low p	0.94	0.97	0.96	0.94	0.89	0.94
High p	1.00	0.98	0.95	0.95	0.99	0.97

Note

Confidence interval coverage for sampling strategies with 5 capture occasions (Occ.).

Confidence interval coverage was always ≥0.89 for all scenarios with 5 sampling occasions (Table 3; Supporting information Tables S6, S7). For scenarios with 10 occasions (Table 4; Supporting information Tables S8, S9) coverage decreased after the 7th–8th occasion. This is because the low number of individuals present at the end of the study (due to a low apparent survival) affects the accuracy of the estimates (Figure 1; Supporting information Figures S1, S2).

**Table 4:** Scenario 1 of simulation (state‐dependent detectability and homogeneous assignment probability)

Confidence interval coverage 10 occasions											
	Occ. 1	Occ. 2	Occ. 3	Occ. 4	Occ. 5	Occ. 6	Occ. 7	Occ. 8	Occ. 9	Occ. 10	Average
Low p	0.98	0.95	0.97	0.96	0.94	0.92	0.84	0.65	0.62	0.64	0.84
High p	1.00	0.99	0.97	0.97	0.95	0.91	0.92	0.85	0.91	0.75	0.92

Note

Confidence interval coverage for sampling strategies with 10 capture occasions (Occ.).

### 3.2 Evaluation of sampling strategies

The performance of model‐based prevalence improved more at higher detectability than with an increasing number of capture occasions. Specifically, in scenario 1 the RMSE and the relative bias approached 0 for both sampling strategies with 5 (Table 1) and 10 capture occasions (Table 2) when detectability changed from low to high. In contrast, in sampling strategies with low detectability, the RMSE and the relative bias of model‐based prevalence decreased less rapidly from 5 to 10 capture occasions (Figure 1, Tables 1, 2). We observed the same pattern both in scenario 2 (Supporting information Figures S1, S2, Tables S2, S3) and in scenario 3, although in the latter the bias was small regardless of the sampling strategy (Supporting information Tables S4, S5).

### 3.3 Case study

The best‐supported model had constant parameters (Table 5). Models with state‐dependent apparent survival, detectability, and probability of assignment were also supported (*Δ*AICc < 2). The models with state‐dependent probability of assignment were not identifiable due to the reduced sample size, and we discarded them (see Supporting information Table S10 for a complete list of fitted models). According to the model‐averaged estimates of parameters (Table 6), model‐based prevalence was consistently higher (range: 0.23–0.53) than naive prevalence (range: 0.20–0.46), with the latter always included in the confidence interval of the former (Table 7).

**Table 5:** Model selection results for the case study on wolf × dog hybridization

Model	n_Par_	Deviance	QAICc	ΔQAICc
*π*(*i*)*φ*(i)*p*(*i*)*δ*(*a*1 + *a*2_fix)	4	183.48	192.16	0
*π*(*i*)*φ*(*f*)*p*(*i*)*δ*(*a*1 + **a**2_fix)	5	181.41	192.44	0.28
*π*(*i*)*φ*(*i*)*p*(*f*)*δ*(*a*1 + *a*2_fix)	5	181.76	192.79	0.63

Note

The notation (.) indicates constant parameters while (state) indicates state‐dependent parameters. *π* = initial state probability, *φ* = survival probability, *p* = detection probability, *δ* = assignment probability, *n*_par_ = number of parameters. The term (*a*1 + *a*2_fix) indicates that we constrained the model to have fixed assignment probabilities after first capture.

**Table 6:** Model‐averaged parameter estimates for the case study on wolf × dog hybridization

Parameter	Estimate	95% C.I.
Initial proportion of individuals in state “Wolf” *π*_*w*_	0.60	0.43–0.76
Detection probability of wolves *p*_*w*_	0.42	0.17–0.67
Detection probability of hybrids *p*_*h*_	0.50	0.26–0.77
Survival probability of wolves *φ*_*w*_	0.72	0.39–0.91
Survival probability of hybrids *φ*_*h*_	0.84	0.46–0.99
Assignment probability *δ*	0.79	0.64–0.89

**Table 7:** Naive and model‐based prevalence of hybrids for the case study on wolf × dog hybridization

Prevalence	Occ. 1	Occ. 2	Occ. 3	Occ. 4	Occ. 5
Naive	0.27	0.33	0.20	0.46	0.27
Model‐based	0.32	0.42	0.23	0.53	0.43
95% CI	0.07–0.55	0.09–0.63	0.06–0.48	0.12–0.72	0.09–0.67

## 4 DISCUSSION

We presented a hidden Markov model to estimate prevalence in wildlife population taking into account the imperfect detectability and uncertainty in individuals’ classification. We compared model‐based and naive prevalence showing that the latter can be severely biased when detectability is state‐dependent, in agreement with Jennelle et al. (2007). In addition, we identified another source of bias in naive prevalence which was related to the state‐dependent probability of assignment. We demonstrated that, if naive prevalence has to be used as a proxy for population prevalence, the burden of proof should be placed on demonstrating homogeneity in detection and assignment probabilities (Jennelle et al., 2007; MacKenzie & Kendall, 2002).

### 4.1 Model assumptions

Our approach provides a framework to statistically test differences in detectability and probability of assignment and take them into account to produce unbiased estimates of prevalence. However, a series of assumptions must be met (Lebreton et al., 1992; Otis et al., 1978): (a) parameter and processes estimated for the marked individuals can be applied to the unmarked ones. In particular, because multievent CR models are conditional on first capture, we assume that capture probability is the same for unmarked and marked individuals in order to obtain abundance estimates, (b) marks do not affect the behavior of the individuals, (c) marks are not lost, and they are correctly recognized, (d) individuals alive in the population at time t have homogeneous detectability and apparent survival probability, (e) individuals are independent from each other, and (f) no births, deaths, emigration, or immigration occur during the capture occasions. The assumption of homogeneous detectability and survival can be relaxed by including different sources of heterogeneity in the model structure (Cubaynes et al., 2010; Pradel, 1993; Pradel, Hines, Lebreton, & Nichols, 1997). In particular when estimating abundance attention must be paid to unaccounted for heterogeneity in capture probabilities, which is known to bias estimates of abundance (Pollock, Winterstein, Bunck, & Curtis, 1989).

### 4.2 Limitations of the model

A potential drawback of our approach lies in convergence issues that might occur when there is a high proportion of uncertain individuals in the sample (Pradel et al., 2007). This problem can be overcome by confirming the state of just a handful of individuals with some error‐free method. Specifically, Pradel et al. (2007) showed that in a case study in which the sex of about 80% of the individuals was uncertain, adding few genetic confirmations (24 individuals over 4,025) greatly improved the shape of the likelihood and hence the convergence of the optimization algorithm. This is particularly relevant because the model does not handle assignment errors, so having a high proportion of uncertain individuals is preferable to taking the risk of making assignment errors to reduce such proportion.

### 4.3 Sampling guidelines

Through the simulations we showed that the precision of model‐based prevalence increased proportionally more by enhancing detection probability than by increasing the number of sampling occasions. The precision of survival estimates increased by the same amount with increasing detectability and number of occasions, confirming that a cost‐effective sampling strategy should maximize detectability within capture occasions instead of sampling more frequently. For a specific study, our simulation framework can be used to determine the best trade‐off to obtain a cost‐effective level of accuracy and precision of parameter estimates (Lieury et al., 2017).

### 4.4 Case study

For the hybridization case study, potential differences in the detectability of parental and admixed individuals generate bias in naive prevalence and can originate from various reasons. For example, differences in vocalization behavior (Derégnaucourt, Guyomarc'h, & Spanò, 2005), migratory tendency (Derégnaucourt, Guyomarc'h, & Belhamra, 2004), and social status (Battocchio, Iacolina, Canu, & Mori, 2017) were documented between admixed parental individuals of different species and may cause differential detectability. In wolf packs, in particular, differences in detectability through scat sampling are related to social status and marking behavior (Cubaynes et al., 2010; Marucco et al., 2009).

Previous hybridization studies on wolves (Godinho et al., 2011; Pacheco et al., 2017) acknowledged that, due to uncertainty of classification, a proportion of backcrosses was assigned to the parental cluster (i.e., wolves), leading to an underestimation of their prevalence in the population. With our approach, a conservative *q*_wolf_ threshold can be used for the parental cluster, greatly reducing the chance of type II errors (erroneously classifying hybrids as parentals), as showed by the higher model‐based prevalence values in the case study (Table 7). These results should be considered as a warning that relying on naive prevalence underestimates the hybridization‐related risks for the conservation of the parental populations.

### 4.5 Model extensions and broader applications of the model

The current model formulation contains two states (“Hybrid” and “Parental”). It might be desirable, however, to further split the “Hybrid” state into two distinct states “Hybrid” and “Backcross,” or to include not just one but both parental species. This can be done by increasing the number of states and corresponding parameters depending on the available data and the power of the genetic tests. However, attention should be paid to avoid over‐parameterization (Gimenez, Choquet, & Lebreton, 2003). Multievent models can quickly become parameter‐rich and thus result in nonidentifiability in studies with small sample sizes.

The Viterbi algorithm has been previously applied in the multievent CR framework to reconstruct the reproductive life of individual roe deers (*Capreolus capreolus;* Rouan et al., 2009). We used the Viterbi algorithm to reconstruct the abundance of individuals in different states. This approach can be used in any case study that requires the estimation of abundance and prevalence of individuals in the presence of imperfect detection and uncertainty in state assignment, for example, the estimation of sex ratios in monomorphic species (Genovart et al., 2012; Pradel et al., 2007). Moreover, the model can be extended to include dynamic states by adding a transition probability matrix in the formulation. Such extension would expand its applications to other fields such as epidemiology (e.g., the estimation of the number of infected individuals in a population; Marescot et al., 2018) or reproductive biology studies (e.g., the number of breeders in a population; Desprez, McMahon, Hindell, Harcourt, & Gimenez, 2013).

## CONFLICT OF INTEREST

None declared.

## AUTHOR CONTRIBUTIONS

NLS, OG, and PC conceived the ideas and designed methodology and led the writing of the manuscript; PC and WR organized and supervised the field data collection and logistics; LM collected the data; NLS and OG constructed the models, performed the simulations, and analyzed the data; RC and EF performed the genetic laboratory and cluster analyses. All authors contributed critically to the drafts and gave final approval for publication.

## Data Availability

Codes of the models, simulations, and data are available from the Dryad Digital Repository (https://doi.org/10.5061/dryad.8g8r675).
