# Supplementary material for: Use of hidden Markov capture–recapture models to estimate abundance in the presence of uncertainty: Application to the estimation of prevalence of hybrids in animal populations
Source: Ecol Evol. 2019 Feb 5;9(2):744–55. doi: 10.1002/ece3.4819 (PMC6362442; doi:10.1002/ece3.4819)
Supplement: Supplementary file 1 [file ECE3-9-744-s001.docx]

# SUPPLEMENTARY MATERIALS

1. **MODEL DETAILS**

1.1 Instruction to fit the model in E_SURGE:

**INPUT DATA CODING**:

We coded the data in the following way (individuals were assigned upon first capture and never again):

Example of coding of encounter histories.

| **Capture history** | **State assignment** |
| --- | --- |
| 10330 | Sure wolf (W) |
| 02330 | Sure hybrid (H) |
| 03303 | Uncertain (H) |

**GEPAT:**

**Initial States**

$$\begin{matrix} \boldsymbol{P} & \boldsymbol{H} \end{matrix}$$

$$\left( \begin{matrix} \pi& 1-\pi\end{matrix} \right)$$

**Transitions**

$$\begin{matrix} \boldsymbol{P} & \boldsymbol{H} & \boldsymbol{D} \end{matrix}$$

$$\begin{matrix} \boldsymbol{P} \\ \boldsymbol{H} \\ \boldsymbol{D} \end{matrix}\left( \begin{matrix} \varphi_{p} & 0 & 1-\varphi_{p} \\ 0 & \varphi_{h} & 1-\varphi_{h} \\ 0 & 0 & 1 \end{matrix} \right)$$

**Events**

$$\begin{matrix} \boldsymbol{0} & \boldsymbol{1} & \boldsymbol{2} \end{matrix}$$

$$\begin{matrix} \boldsymbol{0} & \boldsymbol{1} & \boldsymbol{2} \end{matrix}$$

$$\begin{matrix} \boldsymbol{3} \end{matrix}$$

$$\begin{matrix} \boldsymbol{P} \\ \boldsymbol{H} \\ \boldsymbol{D} \end{matrix}\left[ \begin{matrix} 1-p_{p} & p_{p} & 0 \\ 1-p_{h} & 0 & p_{h} \\ 1 & 0 & 0 \end{matrix} \right] \begin{matrix} \boldsymbol{0} \\ \boldsymbol{1} \\ \boldsymbol{2} \end{matrix}\left[ \begin{matrix} 1 & 0 & 0 & 0 \\ 0 & \delta_{p} & 0 & 1-\delta_{p} \\ 0 & 0 & \delta_{h} & 1-\delta_{h} \end{matrix} \right]$$

**GEMACO:**

**IS:** to (for constant), t (for time)

**TRANSITIONS:** i (for constant), f (for state depndendent)

**EVENTS:**

- Step 1: firste+nexte (for constant), firste+nexte.f (for state depndendent), firste+nexte.t (for time)
- Step 2: a (age effect, where age is time since first capture, this allows for having assignment probability upon first capture ≠ assignment probability on recaptures), a.f (for having assignment probability upon first capture ≠ assignment probability on recaptures and a state effect)

**IVFV:**

**Events**

- **Step 1:** fixed first detection to 1
- **Step 2:** fix assignment probability at age 2 (meaning all assignment probabilities after first capture) to 0

1. **TABLES**

**Table S1.** Detectability (p) and assignment probabilities (δ) used for the three main scenarios of simulation for wolves (w) and hybrids (h). Scenario 1 implies state-dependent detectability and homogeneous assignment probability, Scenario 2 implies homogeneous detectability and state-dependent assignment probability, Scenario 3 implies homogeneous detectability and assignment probability.

|  | Scenario 1 | | Scenario 2 | | Scenario 3 | |
| --- | --- | --- | --- | --- | --- | --- |
| Detectability | High | Low | High | Low | High | Low |
| p_w_ | 0.80 | 0.50 | 0.80 | 0.50 | 0.80 | 0.50 |
| p_h_ | 0.80 | 0.50 | 0.50 | 0.30 | 0.80 | 0.50 |
| δ_w_ | 0.80 | 0.80 | 0.80 | 0.80 | 0.80 | 0.80 |
| δ_h_ | 0.80 | 0.80 | 0.80 | 0.80 | 0.60 | 0.60 |

- 1. **Simulation results for Scenarios 2 and 3**

2.1.1 Root Mean Squared Error and Relative Bias

**Table S2.** Scenario 2 (homogeneous detectability and state-dependent assignment probability). Root mean squared error and relative bias of naive and model-based prevalence for sampling strategies with 5 capture occasions (Occ).

| **Root mean squared error** | | | | | |
| --- | --- | --- | --- | --- | --- |
|  | Occ1 | Occ2 | Occ3 | Occ4 | Occ5 |
|  | Low detectability | | | | |
| Naive | 0.21 | 0.25 | 0.21 | 0.41 | 0.29 |
| Model-based | 0.03 | 0.02 | 0.01 | 0.02 | 0.01 |
|  | High detectability | | | | |
| Naive | 0.31 | 0.26 | 0.26 | 0.23 | 0.15 |
| Model-based | 0.04 | 0.02 | 0.00 | 0.00 | 0.00 |
| **Percent relative bias** | | | | | |
|  | Low detectability | | | | |
|  | -0.05 | -0.05 | -0.05 | -0.06 | -0.05 |
|  | 0.01 | 0.01 | 0.01 | 0.02 | 0.01 |
|  | High detectability | | | | |
| Naive | -0.06 | -0.05 | -0.05 | -0.05 | -0.04 |
| Model-based | 0.02 | 0.01 | 0.00 | 0.00 | 0.00 |

**Table S3.** Scenario 2 (homogeneous detectability and state-dependent assignment probability). Root mean squared error and relative bias of naive and model-based prevalence for sampling strategies with 10 capture occasions (Occ).

| **Root Mean Squared Error** | | | | | | | | | | |
| --- | --- | --- | --- | --- | --- | --- | --- | --- | --- | --- |
|  | Occ1 | Occ2 | Occ3 | Occ4 | Occ5 | Occ6 | Occ7 | Occ8 | Occ9 | Occ10 |
|  | Low detectability | | | | | | | | | |
| Naive | 0.19 | 0.15 | 0.17 | 0.08 | 0.28 | 0.17 | 0.16 | 0.13 | 0.02 | NaN |
| Model-based | 0.10 | 0.06 | 0.05 | 0.06 | 0.01 | 0.00 | 0.00 | 0.00 | 0.01 | 0.01 |
|  | High detectability | | | | | | | | | |
| Naive | 0.27 | 0.31 | 0.23 | 0.22 | 0.19 | 0.12 | 0.12 | 0.19 | 0.13 | 0.05 |
| Model-based | 0.00 | 0.00 | 0.00 | 0.00 | 0.00 | 0.00 | 0.00 | 0.00 | 0.00 | 0.00 |
| **Percent relative bias** | | | | | | | | | | |
|  | Low detectability | | | | | | | | | |
| Naive | -0.04 | -0.04 | -0.04 | -0.03 | -0.05 | -0.04 | -0.04 | -0.04 | -0.01 | NaN |
| Model-based | 0.03 | 0.02 | 0.02 | 0.02 | 0.01 | 0.00 | 0.00 | 0.00 | 0.01 | 0.01 |
|  | High detectability | | | | | | | | | |
| Naive | -0.05 | -0.06 | -0.05 | -0.05 | -0.04 | -0.03 | -0.04 | -0.04 | -0.04 | -0.02 |
| Model-based | 0.01 | 0.01 | 0.00 | 0.00 | 0.00 | 0.00 | 0.00 | 0.01 | 0.00 | 0.00 |

**Table S4.** Scenario 3 (homogeneous detectability and assignment probability). Root mean squared error and relative bias of naive and model-based prevalence for sampling strategies with 5 capture occasions (Occ).

| **Root mean squared error** | | | | | |
| --- | --- | --- | --- | --- | --- |
|  | Occ1 | Occ2 | Occ3 | Occ4 | Occ5 |
|  | Low detectability | | | | |
| Naive | 0.00 | 0.01 | 0.00 | 0.00 | 0.00 |
| Model-based | 0.05 | 0.00 | 0.01 | 0.00 | 0.00 |
|  | High detectability | | | | |
| Naive | 0.00 | 0.00 | 0.00 | 0.00 | 0.01 |
| Model-based | 0.03 | 0.01 | 0.00 | 0.00 | 0.00 |
| **Percent relative bias** | | | | | |
|  | Low detectability | | | | |
| Naive | -0.00 | -0.01 | -0.00 | -0.00 | 0.00 |
| Model-based | 0.00 | -0.00 | -0.01 | -0.00 | -0.00 |
|  | High detectability | | | | |
| Naive | 0.00 | -0.00 | 0.00 | 0.00 | 0.01 |
| Model-based | -0.02 | -0.01 | 0.00 | -0.00 | 0.00 |

**Table S5.** Scenario 3 (homogeneous detectability and assignment probability). Root mean squared error and relative bias of naive and model-based prevalence for sampling strategies with 10 capture occasions (Occ).

| **Root Mean Squared Error** | | | | | | | | | | |
| --- | --- | --- | --- | --- | --- | --- | --- | --- | --- | --- |
|  | Occ1 | Occ2 | Occ3 | Occ4 | Occ5 | Occ6 | Occ7 | Occ8 | Occ9 | Occ10 |
|  | Low detectability | | | | | | | | | |
| Naive | 0.05 | 0.00 | 0.00 | 0.00 | 0.00 | 0.01 | 0.01 | 0.00 | 0.00 | 0.01 |
| Model-based | 0.00 | 0.00 | 0.00 | 0.00 | 0.00 | 0.03 | 0.01 | 0.00 | 0.00 | 0.03 |
|  | High detectability | | | | | | | | | |
| Naive | 0.00 | 0.00 | 0.001 | 0.00 | 0.01 | 0.00 | 0.00 | 0.01 | 0.01 | 0.00 |
| Model-based | 0.03 | 0.01 | 0.00 | 0.00 | 0.00 | 0.00 | 0.00 | 0.00 | 0.00 | 0.00 |
| **Percent relative bias** | | | | | | | | | | |
|  | Low detectability | | | | | | | | | |
| Naive | 0.02 | 0.01 | 0.00 | -0.00 | -0.00 | -0.01 | -0.01 | -0.00 | 0.00 | 0.03 |
| Model-based | -0.00 | -0.00 | 0.00 | -0.01 | -0.01 | -0.02 | -0.01 | -0.01 | -0.00 | -0.02 |
|  | High detectability | | | | | | | | | |
| Naive | 0.00 | 0.00 | 0.01 | -0.00 | 0.00 | -0.00 | -0.01 | -0.01 | -0.01 | 0.00 |
| Model-based | -0.02 | -0.01 | -0.00 | -0.00 | 0.00 | -0.00 | -0.00 | -0.01 | 0.00 | 0.00 |

2.1.2 Confidence intervals coverage

**Table S6.** Scenario 2 (homogeneous detectability and state-dependent assignment probability) for sampling strategies with 5 capture occasions (Occ). Confidence interval coverage.

| **Confidence interval coverage 5 occasions** | | | | | | |
| --- | --- | --- | --- | --- | --- | --- |
|  | Occ1 | Occ2 | Occ3 | Occ4 | Occ5 | Ave |
| Low p | 0.97 | 1.00 | 0.99 | 0.95 | 0.98 | 0.98 |
| High p | 1.00 | 1.00 | 1.00 | 1.00 | 0.99 | 1.00 |

**Table S7.** Scenario 2 (homogeneous detectability and state-dependent assignment probability) for sampling strategies with 5 capture occasions (Occ). Confidence interval coverage.

| **Confidence interval coverage 10 occasions** | | | | | | | | | | | |
| --- | --- | --- | --- | --- | --- | --- | --- | --- | --- | --- | --- |
|  | Occ1 | Occ2 | Occ3 | Occ4 | Occ5 | Occ6 | Occ7 | Occ8 | Occ9 | Occ10 | Ave |
| Low p | 0.97 | 1.00 | 1.00 | 0.97 | 0.97 | 0.93 | 0.90 | 0.88 | 0.81 | 0.70 | 0.91 |
| High p | 0.99 | 1.00 | 1.00 | 1.00 | 1.00 | 0.99 | 0.98 | 0.94 | 0.97 | 0.94 | 0.98 |

**Table S8.** Scenario 3 (homogeneous detectability and assignment probability) for sampling strategies with 5 capture occasions (Occ). Confidence interval coverage.

| **Confidence interval coverage 5 occasions** | | | | | | |
| --- | --- | --- | --- | --- | --- | --- |
|  | Occ1 | Occ2 | Occ3 | Occ4 | Occ5 | Ave |
| Low p | 0.98 | 0.98 | 0.99 | 0.98 | 0.99 | 0.98 |
| High p | 1 | 1 | 1 | 1 | 1 | 1 |

**Table S9.** Scenario 3 (homogeneous detectability and assignment probability) for sampling strategies with 10 capture occasions (Occ). Confidence interval coverage.

| **Confidence interval coverage 10 occasions** | | | | | | | | | | | |
| --- | --- | --- | --- | --- | --- | --- | --- | --- | --- | --- | --- |
|  | Occ1 | Occ2 | Occ3 | Occ4 | Occ5 | Occ6 | Occ7 | Occ8 | Occ9 | Occ10 | Ave |
| Low p | 1.00 | 0.98 | 0.99 | 0.98 | 0.95 | 0.92 | 0.95 | 0.92 | 0.77 | 0.87 | 0.93 |
| High p | 1.00 | 1.00 | 1.00 | 0.98 | 1.00 | 1.00 | 0.99 | 0.98 | 0.97 | 0.95 | 0.98 |

- 1. **Case study results**

**Table S10.** Model selection results for the wolf x dog case study. The notation (.) indicates constant parameters, (state) indicates state-dependent parameters, (time) indicates time-dependent parameters. π = initial state probability, φ = survival probability, p = detection probability, δ = assignment probability, N. Par. = number of parameters. The term (a1+a2_fix) indicates that we constrained the model to have fixed assignment probabilities after first capture. The term (a1+a2_fix).state indicates that the assignment probability is state-dependent. Models with state dependent assignment probability have been dropped from the model averaging because they had non-identifiabe parameters.

| Model | N. Par. | Deviance | QAICc | deltaAICc |
| --- | --- | --- | --- | --- |
| π(i)phi(i)p(i)delta(a1+a2_fix).state | 4 | 183.48 | 192.16 | 0.00 |
| π(i)phi(i)p(i)delta(a1+a2_fix) | 4 | 183.48 | 192.16 | 0.00 |
| π(i)phi(state)p(i)delta(a1+a2_fix) | 5 | 181.41 | 192.44 | 0.28 |
| π(i)phi(i)p(state)delta(a1+a2_fix) | 5 | 181.76 | 192.79 | 0.63 |
| π(i)phi(state)p(i)delta(a1+a2).state | 6 | 179.40 | 192.87 | 0.71 |
| π(i)phi(i)p(state)delta(a1+a2).state | 6 | 181.25 | 194.73 | 2.57 |
| π(i)phi(state)p(state)delta(a1+a2_fix).state | 6 | 181.25 | 194.73 | 2.57 |
| π(i)phi(state)p(state)delta(a1+a2_fix) | 6 | 181.40 | 194.88 | 2.72 |
| π(i)phi(i)p(t)delta(a1+a2_fix) | 7 | 180.63 | 196.63 | 4.47 |
| π(i)phi(state)p(t)delta(a1+a2_fix) | 8 | 178.62 | 197.24 | 5.08 |
| π(i)phi(state)p(t)delta(a1+a2).state | 9 | 176.27 | 197.60 | 5.44 |
| π(t)phi(i)p(i)delta(a1+a2_fix) | 8 | 181.48 | 200.10 | 7.94 |
| π(t)phi(state)p(i)delta(a1+a2) | 9 | 179.11 | 200.45 | 8.29 |
| π(t)phi(i)p(state)delta(a1+a2_fix) | 9 | 179.17 | 200.50 | 8.34 |
| π(t)phi(state)p(i)delta(a1+a2).state | 10 | 176.89 | 201.04 | 8.88 |
| π(i)phi(i)p(t)delta(a1+a2).state | 9 | 180.63 | 201.96 | 9.80 |
| π(t)phi(i)p(i)delta(a1+a2).state | 9 | 180.98 | 202.31 | 10.15 |
| π(t)phi(i)p(state)delta(a1+a2).state | 10 | 178.41 | 202.56 | 10.40 |
| π(t)phi(state)p(state)delta(a1+a2_fix) | 10 | 179.04 | 203.19 | 11.03 |
| π(t)phi(state)p(state)delta(a1+a2).state | 11 | 176.88 | 203.95 | 11.79 |
| π(t)phi(i)p(t)delta(a1+a2_fix) | 11 | 178.63 | 205.71 | 13.55 |
| π(t)phi(state)p(t)delta(a1+a2_fix) | 12 | 176.36 | 206.48 | 14.32 |
| π(t)phi(state)p(t)delta(a1+a2).state | 13 | 173.76 | 207.04 | 14.88 |
| π(t)phi(i)p(t)delta(a1+a2).state | 12 | 178.12 | 208.24 | 16.08 |

## FIGURES

## Simulated prevalence estimates for scenario 2: homogenous detectability and state-dependent assignment probability


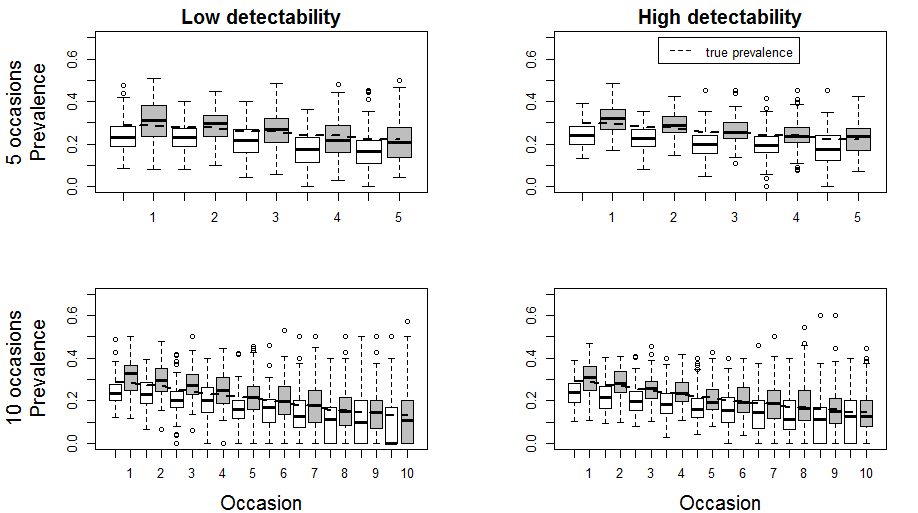


Figure S1. Scenario 2 (homogeneous detectability and state-dependent assignment probability). Sampling strategies with 5 (upper panels) vs 10 (lower panels) capture occasions and low (left-column panels) vs high (right-column panel) detectability. True prevalence is represented as a dashed line while the 100 values of naive and model-based prevalence are displayed in the white and grey boxplots, respectively.

## Simulated prevalence estimates for scenario 3: homogenous detectability and assignment probability


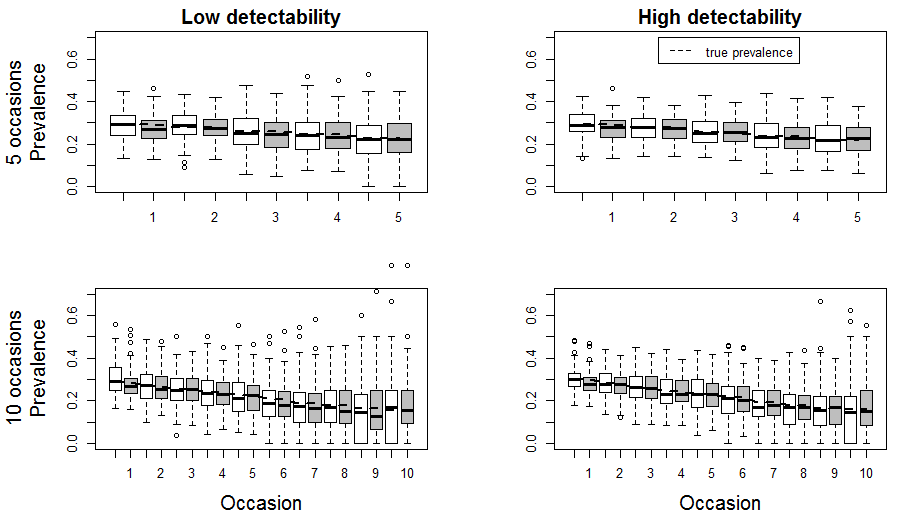


Figure S2. Scenario 3 (homogeneous detectability and assignment probability). Sampling strategies with 5 (upper panels) vs 10 (lower panels) capture occasions and low (left-column panels) vs high (right-column panel) detectability. True prevalence is represented as a dashed line while the 100 values of naive and model-based prevalence are displayed in the white and grey boxplots, respectively.

## Simulated parameter estimates for simulations scenario 2:homogenous detectablity and state-dependent assignment probability


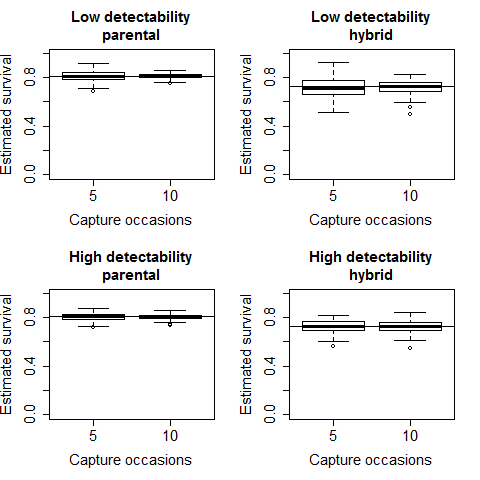


**Figure S3.** Scenario 2 (homogeneous detectability and state-dependent assignment probability). Boxplots of 100 simulated survival estimates for parentals (left two panels) and hybrids (right two panels) for each sampling strategy. Sampling strategies with low detectability are in the top row, sampling strategies with high detectability are in the bottom row. Estimates obtained with sampling strategies with 5 and 10 capture occasions are compared in each panel.


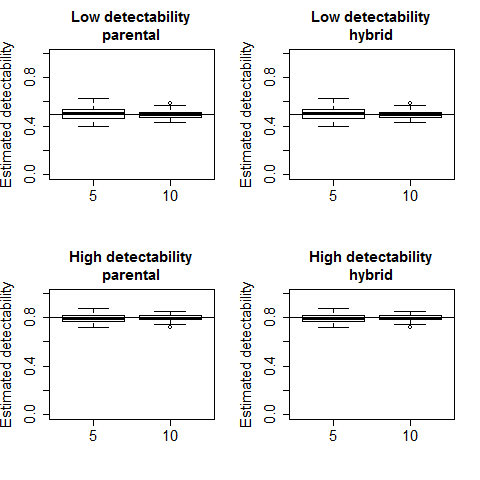


**Figure S4**. Scenario 2 (homogeneous detectability and state-dependent assignment probability). Boxplots of 100 simulated detectability estimates for parentals (left two panels) and hybrids (right two panels) for each sampling strategy. Sampling strategies with low detectability are in the top row, sampling strategies with high detectability are in the bottom row. Estimates obtained with sampling strategies with 5 and 10 capture occasions are compared in each panel.


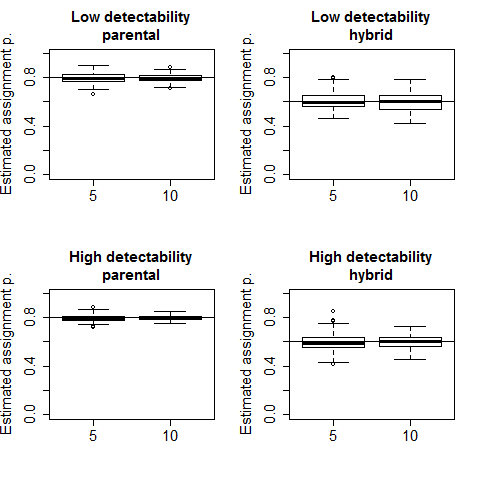


**Figure S5.** Scenario 2 (homogeneous detectability and state-dependent assignment probability). Boxplots of 100 simulated assignment probability estimates for parentals (left two panels) and hybrids (right two panels) for each sampling strategy. Sampling strategies with low detectability are in the top row, sampling strategies with high detectability are in the bottom row. Estimates obtained with sampling strategies with 5 and 10 capture occasions are compared in each panel.


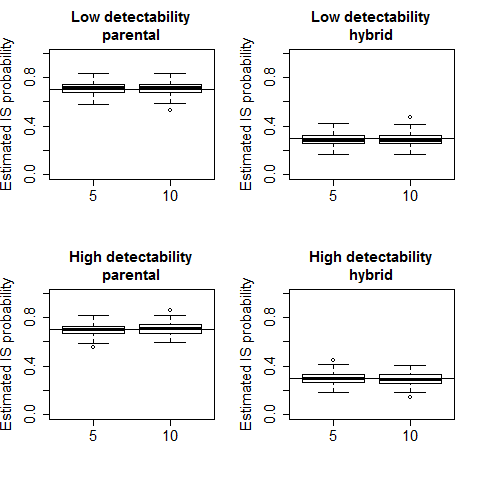


**Figure S6.** Scenario 2 (homogeneous detectability and state-dependent assignment probability). Boxplots of 100 simulated initial state (IS) probability estimates for parentals (left two panels) and hybrids (right two panels) for each sampling strategy. Sampling strategies with low detectability are in the top row, sampling strategies with high detectability are in the bottom row. Estimates obtained with sampling strategies with 5 and 10 capture occasions are compared in each panel.

## Simulated parameter estimates for simulations scenario 3:homogenous detectablity and assignment probability


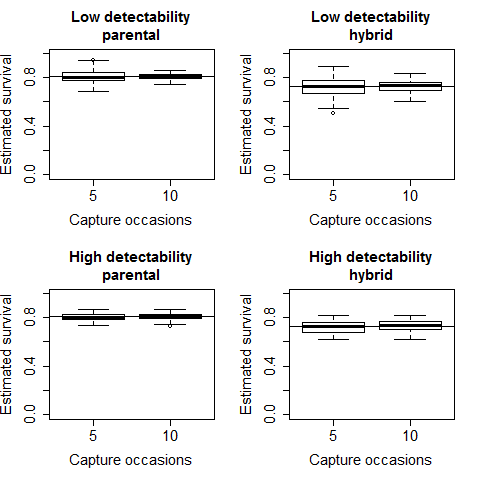


**Figure S7.** Scenario 3 (homogeneous detectability and assignment probability). Boxplots of 100 simulated survival estimates for parentals (left two panels) and hybrids (right two panels) for each sampling strategy. Sampling strategies with low detectability are in the top row, sampling strategies with high detectability are in the bottom row. Estimates obtained with sampling strategies with 5 and 10 capture occasions are compared in each panel.


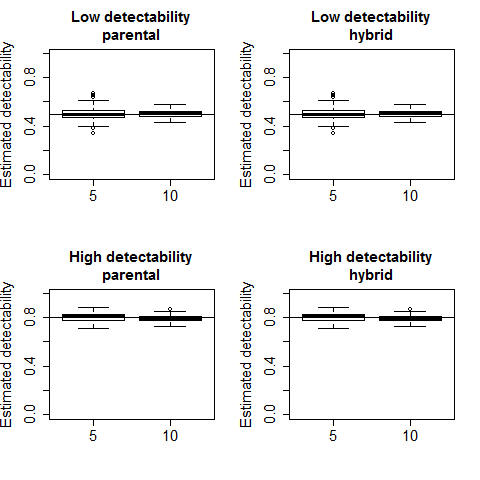


**Figure S8.** Scenario 3 (homogeneous detectability and assignment probability Boxplots of 100 simulated detectability estimates for parentals (left two panels) and hybrids (right two panels) for each sampling strategy. Sampling strategies with low detectability are in the top row, sampling strategies with high detectability are in the bottom row. Estimates obtained with sampling strategies with 5 and 10 capture occasions are compared in each panel.


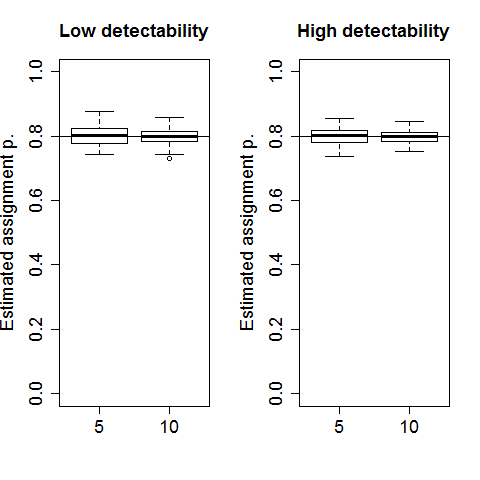


**Figure S9.** Scenario 3 (homogeneous detectability and assignment probability). Boxplots of 100 simulated assignment probability estimates. Sampling strategies with low detectability are on the left panel, sampling strategies with high detectability are on the right panel. Estimates obtained with sampling strategies with 5 and 10 capture occasions are compared in each panel.


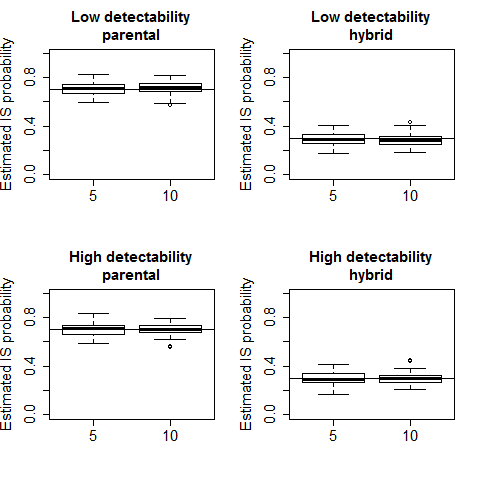


**Figure S10.** Scenario 3 (homogeneous detectability and assignment probability). Boxplots of 100 simulated initial state probability estimates for parentals (left two panels) and hybrids (right two panels) for each sampling strategy. Sampling strategies with low detectability are in the top row, sampling strategies with high detectability are in the bottom row. Estimates obtained with sampling strategies with 5 and 10 capture occasions are compared in each panel.
